# Probiotics as Antiviral Agents in Shrimp Aquaculture

**DOI:** 10.1155/2013/424123

**Published:** 2013-04-30

**Authors:** Bestha Lakshmi, Buddolla Viswanath, D. V. R. Sai Gopal

**Affiliations:** Department of Virology, Sri Venkateswara University, Tirupati 517502, India

## Abstract

Shrimp farming is an aquaculture business for the cultivation of marine shrimps or prawns for human consumption and is now considered as a major economic and food production sector as it is an increasingly important source of protein available for human consumption. Intensification of shrimp farming had led to the development of a number of diseases, which resulted in the excessive use of antimicrobial agents, which is finally responsible for many adverse effects. Currently, probiotics are chosen as the best alternatives to these antimicrobial agents and they act as natural immune enhancers, which provoke the disease resistance in shrimp farm. Viral diseases stand as the major constraint causing an enormous loss in the production in shrimp farms. Probiotics besides being beneficial bacteria also possess antiviral activity. Exploitation of these probiotics in treatment and prevention of viral diseases in shrimp aquaculture is a novel and efficient method. This review discusses the benefits of probiotics and their criteria for selection in shrimp aquaculture and their role in immune power enhancement towards viral diseases.

## 1. Introduction

Aquaculture is a worldwide activity and considered as a major economic and food production sector as it is an increasingly important source of protein available for human consumption. According to FAO, the supplies of fish, crustaceans, and molluscs from aquaculture increased from 3.9% of total production by weight in 1970 to 27.3% in 2000, and aquaculture is growing more rapidly than all other animal-food-producing sectors [1]. In Europe itself, it is estimated that aquaculture production will exceed 2.5 million tonnes by 2015 and reach 4 million tonnes by 2030 [1]. Aquaculture production is heavily dominated by China and other developing countries in the Asia Pacific region, which accounts for 89% by volume [2]. Intensification of aquaculture had led to the promotion of conditions that favor the development of a number of diseases and problems related to biofouling. Shrimp farming is an aquaculture business; that is, it exists in either a marine or freshwater environment, producing shrimp or prawns. Over the past five years, there have been major developments in shrimp farming. 

Diseases are primary constraint to the growth of many shrimp species, which are exposed to stressful conditions, adverse environmental conditions. Consequently, wide ranges of chemicals particularly antimicrobial agents are used in shrimp farming to prevent and to treat diseases. The usage of these antimicrobial agents has increased enormously and tonnes of antibiotics are distributed in the biosphere during an antibiotic era of only about 60-year duration [3]. In the United States alone, 18,000 tonnes of antibiotics are produced each year for medical and agriculture purposes; 12,600 tonnes are used for the nontherapeutic treatments of livestock in order to promote growth [3]. In the last 20 years, there has been a fourfold growth in aquaculture industries worldwide [4–6]. This impressive industrial development has been accompanied by some practices that are potentially damaging to human and animal health [4, 7] which include passing of large amount of veterinary drugs into the environment [8, 9]. For instance, aquaculture of shrimp and salmon has been accompanied by an important increase in the use of prophylactic antibiotics in the aquatic environments [10–12]. The emergence of antibiotic-resistance among shrimp pathogens undermines the effectiveness of the prophylactic use of antibiotics in aquaculture [13, 14] and increases the possibilities for passage not only of these antibiotic-resistant bacteria but also of their antibiotic resistance determinants to bacteria of terrestrial animals and human beings, including pathogens. Another problem created by the excessive use of antibiotics in industrial aquaculture is the presence of residual antibiotics in commercialized fish, shrimp, and shellfish products [10, 14–19]. In shrimp farming, the passage and permanent existence of large amounts of antibiotics in the environment of water and sediments also have the potential to affect the presence of the normal flora and plankton in those niches, resulting in shifts in the diversity of the microbiota [20–22].

In this view, the development of nonantibiotic agents for health management in shrimp farming is necessary. Probiotics were known to control pathogens through a variety of mechanisms; hence, they are exploited as an alternative to antibiotic treatment. The review mainly focuses on the benefits of probiotics in shrimp farming especially against viral diseases. It also illustrates different methods to study the antiviral activity of probiotics with a brief explanation of shrimp immune system and its antiviral immune response.

## 2. Viral Diseases of Shrimp

Shrimp aquaculture is the most valuable marine aquaculture industry. Despite the explosive growth in world production of cultivated shrimp, there have also been staggering, periodic losses due to diseases. Hence, diseases are now considered as one of the critical limiting factors in the shrimp culture. Serious viral disease outbreaks of shrimp challenge the shrimp industry to be better prepared in the view of a broadened knowledge about shrimps and their pathogens so that disease prevention methods could be improved. This requires shifted awareness to biosecurity, that is, possible methods of cultivating shrimp in restricted systems designed to prevent the entry of potential pathogens. The industry also realized that a good number of disease outbreaks originated from careless transboundary movement of contaminated but grossly normal aquaculture stocks. Estimates of economic losses suggest that developing countries in Asia lost at least US$1.4 thousand million due to diseases in 1990 alone. Since then, losses have increased. Reports from China suggest that losses in 1993 one of US$1 thousand million due to shrimp viral disease outbreaks (ADB/NACA, in press). A 1995 estimate suggests that aquatic animal disease and environment-related problems may cause annual losses to aquaculture production in Asian countries of more than US$3 thousand million per year (ADB/NACA, in press). According to a recent World Bank report, global losses due to shrimp disease are around US$3 thousand million, and the Bank recommends the investment of US$275 million in shrimp disease research over the next 15 years [23]. Some of major viral diseases that affect various species of shrimp are shown in the Table 1.

## 3. Traditional Methods of Disease Control in Shrimp Aquaculture

Currently, there are advanced molecular techniques available for detection and control of diseases in shrimp aquaculture. Here are some traditional methods followed from long back, that is, before the molecular techniques are available. Intensive and superintensive culture systems will become more common and will compete well with traditional methods.

### 3.1. Use of Postlarvae (PL)

The postlarvae (PL) were extensively used by the first shrimp cultivation systems. These were collected from the tidal flow or hand collection from nearby geographical areas. Primarily, the stocking densities were low; hence the disease problems were also low, but the production was also relatively low. As the demand for shrimp increased after 1980 the stocking density gradually increased which caused an increase in production volume and relative increase in disease problems. The solution for this is to select the PL, which are labeled as specific pathogen free (SPF). The seed before farming is diagnosed for the presence of disease and certified as pure, and then they are supplied to farmers.

### 3.2. Disease Management

Regarding the disease management, the essential factors to be considered are treatment of wastewater, sludge, disposal of diseased dead shrimps from ponds, and postharvest processes. Usually farmers in Asian countries release wastewater without treatment, that is, direct disposal of dead or diseased shrimp which is not a good practice. The farmer must be sure that the wastewater and effluent are free of pathogens. The direct disposal of the diseased dead shrimp causes the transmission and spread of the disease. Horizontal transmission of white spot syndrome virus (WSSV) through water and feeding of infected shrimps and movement of infected live animals have been known to be a probable route for the spread of the disease [61].

### 3.3. Effluent Management

Different procedures were applied for treatment of wastewater and effluent. Fishes like tilapias and milkfish were reared in the settlement ponds as biological filters, and the discharge water was released into the settlement ponds for some time before releasing into the open water. To reduce the negative impact of effluents, the use of effective microorganism product was suggested where the microorganisms do the recycling of sludge and used it as fertilizer. Usually the farmers dry the pond for a period of one to two months, then plough and turnover the sludge at the pond bottom, and carry out the disinfecting, drying, and flushing methods to ensure that the dark smelly pond bottom is cleaned and made suitable for shrimp cultivation [62]. Recycling of sludge and providing settlement ponds are some of the approaches recommended to mitigate shrimp pond effluents [63].

### 3.4. Phage Therapy

The use of bacteriophage in the control of bacterial population is not a new science, but this application in shrimp hatcheries is recently much focused. Phages are obligate intracellular parasites, which have no intrinsic metabolism and require the metabolic machinery of the host cell to support their reproduction. They subsist on the bacterial cells, lead lytic and lysogenic life cycle, and make the survival of host extremely difficult. They are species specific, self-perpetuating, and genetically flexible in nature. Bacteriophages are highly abundant in the aquatic environment ranging from 10^4^ mL^−1^ to in excess of 10^8^ mL^−1^. Numbers are typical 3–10 times greater than the bacterial counts although there is substantial variation between ecosystems. Bacteriophages specific to *Vibrio harveyi* isolates were isolated from oyster tissue and shrimp hatchery water lysed 70% of the *V. harveyi* isolates tested. In hatchery trials bacteriophage treatment at 2 × 10^6^ pfu/mL showed a 85% survival of *Penaeus monodon* compared to the control and antibiotic treated ponds [64]. Good numbers of phages have also been isolated against important bacterial fish pathogens. Thus, these bacteriophages could be used as biocontrol agents in the shrimp hatcheries.

### 3.5. Use of Chemicals

Sodium hypochlorite, EDTA, ortho-toluidine, sodium thiosulfate, iodine-PVP formalin, caustic soda (NaOH) and chlorine liquid, Treflan, and muriatic acid are some of the chemicals that are commonly used in various steps of shrimp cultivation. The pesticides that are frequently used in shrimp farming are organochlorines (endosulfan), organophosphates (azinphosethyl, chlorpyrifos, diazinon, dichlorvos, malathion, monocrotophos, parathion, and trichlorfon), carbamates (carbaryl), and others including paraquat, rotenone, nicotine, copper sulphate, formalin, trifluralin, and butachlor.

### 3.6. Use of Antimicrobial

Oxytetracycline (mixed in feed) is the most commonly used antimicrobial and used in combination with chloramphenicol, oxolinic acid, and formalin. Other antibiotics used in shrimp farming are sulfonamides, fluoroquinolones, nonfluorinated quinolones, tetracyclines, chloramphenicol, gentamicin, trimethoprim, and so forth.

## 4. Probiotics in Shrimp Aquaculture

The use of probiotics in humans is a success. The conventional definition of probiotics is “live microorganisms when added to food help to reconstruct a balanced indigenous microflora in the GIT of host” [65–68]. An expert with the Joint Food and Agriculture Organization (FAO/WHO) stated that probiotics are live microorganisms, which, when consumed in adequate amounts, confer a health benefit for the host [69]. While coming to define the probiotics for aquaculture, some specific factors are to be considered. It can be assumed in aquaculture that the intestinal microbiota does not exist as entity by itself but that there is a constant interaction with the environment and the host functions. Considering all these, Verschuere et al. [70] proposed a modified definition of a probiotic as “a live microbial adjunct which has a beneficial effect on the host by modifying the host-associated or ambient microbial community, by ensuring improved use of the feed or enhancing its nutritional value, by enhancing the host response towards disease, or by improving the quality of its ambient environment.” Generally, probiotic strains have been isolated from indigenous and exogenous microbiota of aquatic animals. Gram-negative facultative anaerobic bacteria such as* Vibrio* and *Pseudomonas* constitute the predominant indigenous microbiota of a variety of species of marine shrimp [71].

In contrast to saltwater fish and shrimp, the indigenous microbiota of freshwater species tends to be dominated by members of the genera *Aeromonas*, *Plesiomonas*, representatives of the family *Enterobacteriaceae,* and obligate anaerobic bacteria of the genera *Bacteroides*, *Fusobacterium, *and *Eubacterium* [72]. Lactic acid producing bacteria are generally sub-dominant in GUT and represented essentially by the genus *Carnobacterium* [73]. In aquaculture, however, *Vibrio *spp., *Bacillus* spp., lactic acid bacteria, and microalgae are mainly utilized as probiotics for growth and survival enhancement and reduction of pathogen [30, 74–79]. The significance of these microbiota is listed in Table 2. They have gained acceptance as being more effective than administering antibiotics or chemical substances. More recently, beneficial microbes for aquaculture have been isolated from seawater, sediment, and gastrointestinal tract of aquatic animals that have the ability to produce substances that inhibit pathogens [80–84].

### 4.1. Criteria for Selection of Probiotics for Shrimp Aquaculture

**Figure 1:**
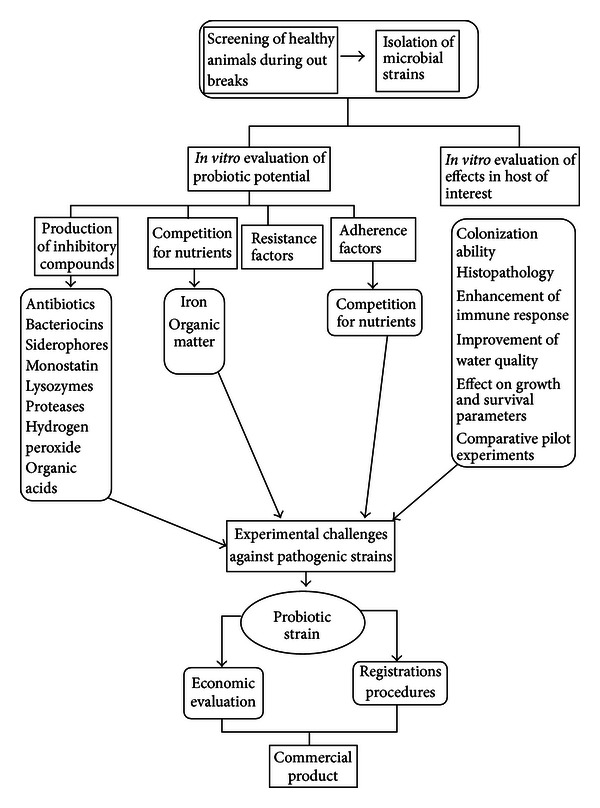
Procedure for evaluation of probiotic potential of microbial strain for shrimp aquaculture.

For the consideration of a microbial strain as a probiotic and for its application in shrimp farming it should be evaluated in a systematic scientific method. Stepwise procedure for the evaluation of the probiotic potential of a microbial strain and its application in shrimp farming is shown in Figure 1. Once the strain has been successfully evaluated by the above procedure, it can be assured as a potential probiotic strain and can be safely applied in the shrimp aquaculture.

### 4.2. Evaluation of Probiotic Potential of Microbial Strains Other Than Animal Origin

**Figure 2:**
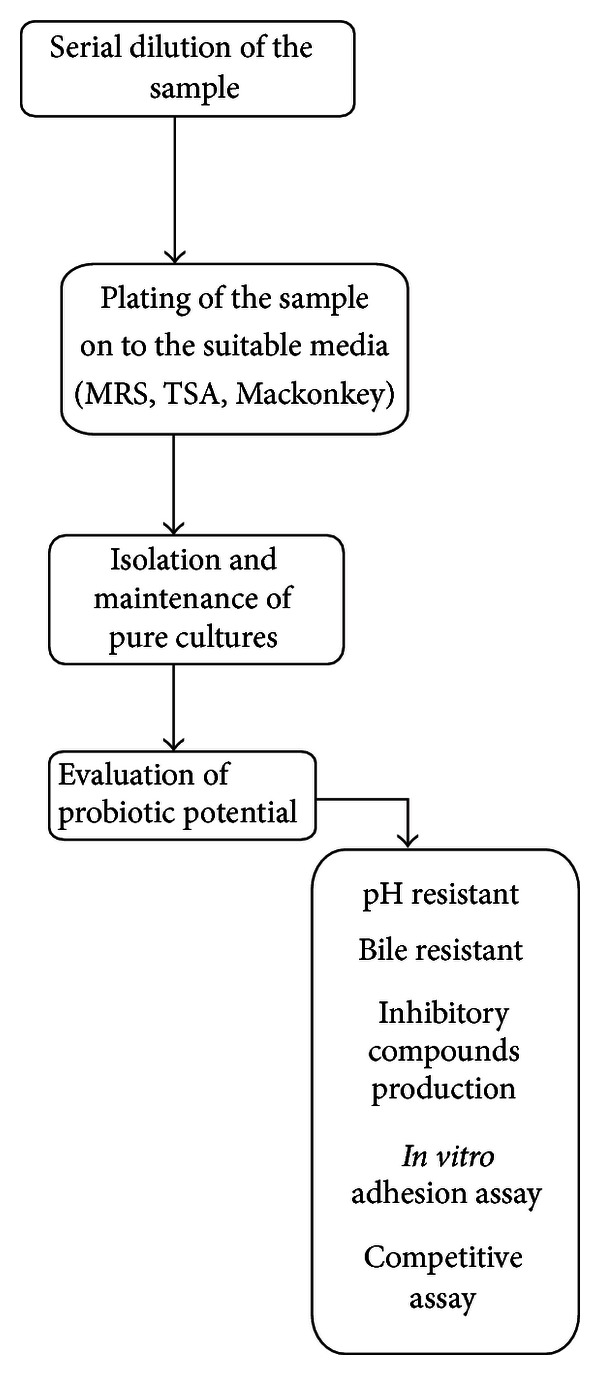
Microbiological procedure for evaluation of probiotic potential of microbial strain.

Some of the probiotic strains are isolated from fermented foods, pond sediments, soil, water, and so forth. The procedure for evaluation of probiotic potential of microbial strains other than animal origin is illustrated in Figure 2. The experimental conditions of the probiotic potential tests vary according to the target host and the further application of probiotics. After the above evaluation process, the strain is further tested for economic evaluation.

### 4.3. Application of Probiotics in Shrimp Aquaculture

Probiotic activity is mediated by a variety of effects that are dependent on the probiotic itself, the dosage employed, treatment duration and route, and frequency of delivery. Some probiotics exert their beneficial effects by elaborating antibacterial molecules such as bacteriocins that directly inhibit other bacteria or viruses, actively participating in the fight against infections, whereas others inhibit bacterial movement across the gut wall (translocation), enhance the mucosal barrier function by increasing the production of innate immune molecules, or modulate the inflammatory/immune response. Several studies have demonstrated that pattern recognition receptors (PRPs), such as toll-like receptors (TLRs) signaling pathways, immune responses, and the secretion of antimicrobial peptides such as defensins and chemokines by the epithelium play important roles in these mechanisms [85, 86]. 

Here are some reports, which stand as evidence for the beneficial effects of Probiotics. *Bacillus *S11, previously isolated from the GIT of *P. monodon* brood stock caught in the gulf of Thailand, demonstrated effective probiotic protection with *P. monodon* [30]. After a 100-day feeding trial with probiotic supplemented and nonsupplemented (control) feeds, *P. monodon* (from PL30 onwards) exhibited significant differences in growth, survival, and external appearance between the two groups. After challenging shrimps with a shrimp pathogen, *Vibrio harveyi* by immersion for 10 days, all probiotic treated groups had 100% survival, whereas the control group had only 26% survival which suggested competitive exclusion by probiotic *Bacillus* S11. Probiotic *Bacillus subtilis* UTM 126 was known to produce antimicrobial activity against vibriosis in juvenile shrimp, *Litopenaeus vannamei* [32].

Consideration of bacterial strains selected as probiotics should be safe to use as biological control. The *extent of safe use of the* microbes that have been used traditionally in probiotics can be confirmed through a long period of experience [87]. Yasuds and Taga [88] suggested that probiotic bacteria would be found to be useful not only as food but also as biological controllers of fish disease and activators of nutrient regeneration. The biological control in aquaculture emerges and since then the research effort has continuously increased. Generally, bacteria play two major roles as beneficial bacteria and pathogenic forms; beneficial bacteria are helpful in nutrient recycling and organic matter degradation and thus clear the environment [89]. Pathogenic bacteria are the causative agents of bad water quality, stress, and diseases as they act as primary and secondary pathogens [90].

The inhibitory effects of *Bacillus *sp. may be due to the production of antibiotics, bacteriocins, lysozymes, proteases, and hydrogen peroxide and the alteration of pH values by the production of organic acids [70]. Probiotics also influence the immune system of the fish, shrimp, and other aquatic species. *Streptomyces* has been applied as a probiotic in the laboratory culture of *Penaeus monodon*, which showed better water quality parameters than the control tank and increased length and weight in terms of growth [33]. Some probiotic products like Super-biotic, Super Ps, Zymetin, and Mutagen [91] were reported to play a vital role in postlarvae of *P. monodon* by maintaining good water quality parameters throughout the culture period. It was reported that *Bacillus subtilis* E20, isolated from the human health food, was used for white shrimp *Litopenaeus vannamei* larvae where it showed a significant decrease in the cumulative mortality and also increased gene expression of prophenoloxidase I, prophenoloxidase II, and lysozyme of larvae [34].

The above reports suggest that for the immune power and disease resistance enhancement the probiotics need to be given at regular intervals throughout the culture period and any halt in the probiotic supplementation leads to the untreated conditions and the animal become susceptible to the diseases. The benefits of probiotics were summarized in Table 3.

### 4.4. Probiotics in Activation of Shrimp Immune Defences

Probiotics were successfully reported for their beneficial effects in warm-blooded animals. Experiments indicate that probiotic bacteria administered orally may induce increased resistance to enteric infections [92]. As mentioned earlier, shrimp has a poorly developed immune system and probiotics were known to play an important role in the enhancement of immune response in shrimp. The probiotic bacteria *Lactobacillus plantarum* was reported to enhance the immune responses and gene expression in white shrimp, *Litopenaeus vannamei, *when given in diet. The bacteria influenced both the cellular and humoral immune defences in the shrimp. *L. plantarum* was known to enhance the phenoloxidase (PO) activity, prophenoloxidase (ProPO) activity, respiratory bursts, superoxide dismutase (SOD) activity and clearance efficiency of *Vibrio alginolyticus*, peroxinectin mRNA transcription, and survival rate after challenge with *V. alginolyticus*. These effects were observed when the bacteria was given in the diet at 10^10^ cfu (kg diet)^−1^ for 168 hrs [93]. 

The *Lactobacillus plantarum* was also effective on *Vibrio harveyi*. On experimental challenge with *V. harveyi, Litopenaeus vannamei* showed increased resistance when compared to the control group. This was because the probiotic strain showed an immune reactive effect on the host. The probiotic bacteria are known to produce extracellular compounds that can stimulate the nonspecific immune response. The *L. plantarum* was responsible for the increase in total haemocyte count and phenoloxidase activity. In this report, the most effective elimination of the haemolymph and the hepatopancreas by the shrimp fed with the probiotic-supplemented diet could be related to the elevated agglutinating activity [31].

Probiotic *Pediococcus acidilactici* showed an effect on antioxidant defenses and oxidative stress of *Litopenaeus vannamei *when challenged with *Vibrio nigripulchritudo*. It was effective on the antioxidant defenses like SOD, catalase (CAT), glutathione peroxidase, total antioxidant status (TAS), glutathiones, and induced tissue damage. The probiotic strain was efficient in maintaining the antioxidant defence levels for a longer period than the control and uninfected groups [94]. This suggests that the probiotic bacteria besides enhancing the immune defenses also maintain the defence levels in the shrimp offering a prolonged protection. Probiotics strains *Vibrio* P62, *Vibrio* P63, and *Bacillus* P64 were isolated from hepatopancreas of healthy wild shrimp *Penaeus vannamei,* and their immunostimulatory effect was studied. Among the three, P64 showed a significantly higher immunity index and showed immune response similar to that of *V. alginolyticus* whereas the other two only showed good probiotic properties. Here, the P64 gave the immune alert with a significant increase in the hyaline cell population [28].

Some *Vibrio* spp. were assessed for their probiotic potential for *L. vannamei*. Among the species tested *V. alginolyticus* (NCIMB 1339) and *V. gazogenes* (NCIMB 2250) showed antagonistic activity towards shrimp pathogens vibriosis. When the juvenile shrimps were fed with chitin and *V. gazogenes,* they caused a significant decline in the number of vibrio-like bacteria in the fore and hind gut. In this study, the *Vibrio* and chitin mixture caused significant changes in haemocyte numbers. This change in haemocyte number probably reflects the immunological status of shrimp because these are involved in both cellular and humoral defences of the shrimp [95].

## 5. Probiotics for Viral Diseases

As mentioned earlier, probiotics have the capability of enhancing the immune response in fish and shrimp. For the protection from viral diseases, no specific drug was designed; besides the use of antibiotic is giving rise to a new type of resistant strains. Enhancement of the disease resistance in animal and the development of the immune power are the best option to prevent and resist the viral infections. For this purpose, one needs to have a proper understanding of the immune system and the type of immune response in the animal.

### 5.1. Outlines of the Immune System of Shrimp

**Figure 3:**
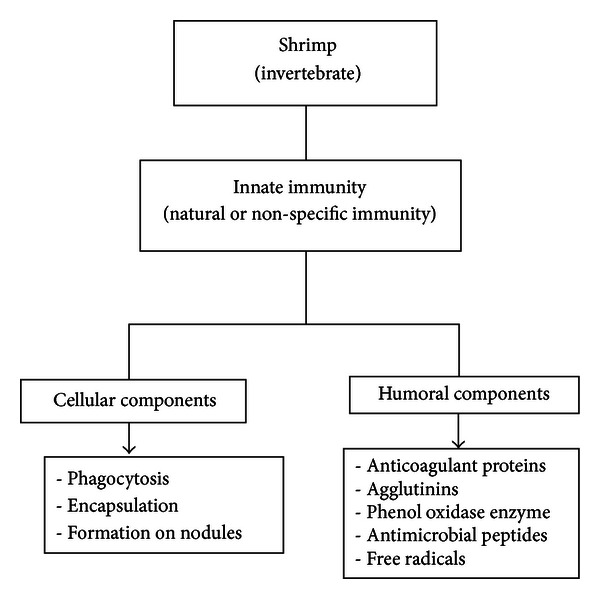
Outline of immune defense system in shrimp.

Fish and shrimp differ significantly in their ability and the degree to which they respond to immune challenge. Shrimps have a poorly evolved defense mechanism and the capacity to recognize, expand the specific recognition, express specific recognition, and coordinate defense is much lower in shrimp when compared to fish. They do not have the ability to produce immunoglobulins; that is, adaptive memory is completely absent, and so they totally depend only on innate immune system [96]. The innate immune system includes both the humoral and cellular components which work in co-ordination with each other for the detection and elimination of all foreign organisms potentially hazardous for the host [97]. The cellular and humoral components are illustrated in Figure 3.

There was a report on the passive immunization of the tiger prawn, *Penaeus monodon*, using rabbit antisera to *Vibrio harveyi* [98]. In this method, the extracellular product of bacterium, *V. harveyi* strain 820514, has been isolated and it was carried on for purification process and injected into the rabbit for production of antiserum. The antiserum was collected from the rabbit and was injected intramuscularly into the prawn with an interval of 10 days and 17 days. When compared to the control ponds, the experimental ponds showed the postponement of the mortality for 2 weeks after postbacterial challenge. It was concluded that the protection given by this passive immunization is a relatively short-lived one. 

As mentioned earlier, shrimps or prawns lack a specific defense system, but the later research and reports suggest that specific immunity can be induced in the shrimp. Oral vaccination of the shrimp, *P. monodon,* towards WSSV showed a significant decrease in the mortality rate when compared to the control group [99]. In this experiment, inactivated bacteria overexpressing the WSSV envelope proteins VP19 and VP28 coated on food pellets were selected for delivery of the WSSV proteins. After vaccination, it was observed that vaccination with either VP28 alone or a mixture of VP28 and VP19 resulted in lower mortalities of 30% and 50% compared to the group vaccinated with the empty vectors. It was also reported that the DNA vaccines encoding viral envelope proteins confer protective immunity against WSSV in black tiger shrimp [100]. But further studies like comparison of immune response between the subunit vaccines and DNA vaccines, immune response towards enveloped, and nonenveloped virus are necessary.

### 5.2. Antiviral Immune Response in Shrimp

Antiviral immune response in shrimp is mediated through the pattern recognition receptors (PRRs). When a virus enters into the shrimp, the infected cells contain viral components like genomic DNA and RNA or dsRNA, and these viral components are sensed by means of PRRs. These PRRs are known to trigger effective and appropriate antiviral responses, including production of various cytokines and induction of inflammatory and adaptive immune reactions [101]. Antiviral immune responses include many antiviral-related proteins/genes, antibody or immune stimulants mediated antiviral activity, cytokine-activated mediated antiviral response, apoptosis [102], phagocytosis, and prophenoloxidase system (Table 4).

In a comparative study of the efficiency of antiviral immune responses including phagocytosis, apoptosis, and ProPO system in the shrimp *Marsupenaeus japonicas, * it was reported that the phagocytosis and apoptosis play a major role in the antiviral immune response whereas the ProPO system is of only minor part [103]. The viral envelope proteins play very important role in the mechanism of infection of virus. It was documented that the viral proteins VP68, VP281, and VP466 have a very significant role in the infection of WSSV [104–106]. The VP466 is known to enhance the innate host immune response in shrimp. It acts as a GTPase-activating protein and forms a complex with the host shrimp Rab and increases its GTPase activity *in vivo* and *in vitro*. The complex induced rearrangements on the actin cytoskeleton, resulting in the formation of actin stress fibers which promoted the phagocytosis against virus [107]. This idea paves the way for the application of viral proteins as the immune enhancers in the host (shrimp) and can also be employed in the development of subunit vaccines.

Another mechanism in the antiviral immune response is the RNA interference (RNAi) method, which has been applied to silence viral genes in eukaryotic organisms. It was reported that a specific 21 bp short interfering RNA (VP28-SiRNA) was designed targeting a major envelope gene VP28 of WSSV. It readily silenced the transcription and translation of the viral gene and significantly reduced the mortality rate of the shrimp [108]. Thus, the RNAi mechanisms stand as an effective therapeutic strategy for viral infections in shrimp.

### 5.3. Study of Antiviral Activity of Probiotics

The exact and accurate methods for the study of antiviral activity of probiotics were not mentioned. From the earlier reports of Kamei et al. [109] and Direkbusarakom et al. [110], the antiviral activity was studied by plaque assays. In this plaque assay the bacterial cultures and the virus were mixed in equal volumes at regular intervals. From this mixture, the aliquots were withdrawn for plaque assay. This mixture was added to the cell cultures, and the antiviral activity was investigated from the rate of plaque reduction. The rate of plaque reduction was figured out by the following formula:

(1) P.F.U.  +0  time−P.F.U. at  each  reaction  timeP.F.U.  at  0  time×100%.

From the plaque reduction assay the bacterial strains *Pseudomonas*, *Aeromonas*/*Vibrio*, and *Coryneforms* were reported for their antiviral substances [109]. A novel eukaryotic cell culture model was proposed to study the antiviral activity of potential probiotic bacteria [111]. This model was successfully applied for human virus. This method is based on the mechanism of probiotic bacteria by which they fight infections for exclusion of pathogens by means of competition for attachment and stimulation of host cell immune defenses [112]. The approved model of this study has not been established yet in aquaculture. With some modifications, this cell culture model can be applied for aquaculture. The antiviral assays in this cell culture model include the following.

- *Pretreatment of cells with bacteria,* where the monolayers/cell lines were first incubated with viable probiotic bacteria and the unbound bacteria were washed off. Now monolayers/cell lines were challenged immediately with the virus and incubated. The cell survival was considered for the antiviral activity.
- *Coincubation of bacterial and virus (competition assay).* In this method, the viable probiotic bacteria and virus were added simultaneously to the cell lines and further incubated under prescribed conditions. Dose dependence between the titer of the virus and number of bacteria was assessed for antiviral activity.
- *Virus adsorption to the bacteria* includes the coincubation of the viable probiotic bacteria with the virus. Then, the bacteria were removed by centrifugation, and the pellet was prepared for immunofluorescence. Residual viral infectivity in supernatants was assayed on cell lines for comparing the TCID50 to the inoculum titer. The virus alone was treated in the same way as the control.
- *Antiviral effect of bacterial supernatants:* Here the supernatants of the viable probiotic bacteria (after inoculation and incubation) were collected and were added on the cell lines/monolayer as incubation medium followed by immediate virus challenge. For the antiviral activity, the CPE was determined after incubation. For the application of the above, the method of aquaculture is important, and it is also important to consider the physical and biochemical factors of the probiotic bacteria and the virus chosen. It is also necessary to have a thorough knowledge of the probiotic bacteria and virus that are being tested.

Generally the virus in the laboratory is cultured in the suitable cell lines, and the virus growth was confirmed from the CPE studies. For example, Taura syndrome virus is cultivated in primary hemocyte culture of shrimp (*Penaeus vannamei*) [113]. The virus growth was confirmed and the titer was considered by studying CPE of the shrimp primary hemocyte culture at regular intervals of 6 hrs, 12 hrs, and 48 hrs. In the same way, the viable probiotic bacteria can be added to these cell lines in any way as mentioned in the cell culture model and CPE can be studied for the antiviral activity of probiotic bacteria.

Real-time PCR was conducted to measure WSSV copies in shrimp [103]. In this method, the results (Ct value) of viral quantity would be converted into copy number of virus based on the standard curve. Attempts are being made for the application of quantitative real-time PCR in the study of antiviral activity of probiotic bacteria, and the process is under progress. Quantitative real-time PCR is the advanced molecular technique, which is applied to the study of the virus titer. Shrimp mortality assay [103] is a physical method to study the antiviral activity of probiotic bacteria where the survival rate of the shrimp is considered as the parameter for the antiviral activity. This survival rate was considered by comparing with the control ponds and the experimental ponds.

Disease problems and environmental issues in shrimp farming have caused worries about the sustainability of traditional farming practices [114]. Certified, disease-free postlarvae and pond preparation were recognized as two of the most important steps in disease prevention. Several measures had been applied in health management to reduce disease, which included postlarvae selection, specific pathogen free brooders, closed systems, recirculation systems, probiotic application, and some form of biosecurity. Farmers reported that the use of probiotic base products and vitamins is helpful for health management and for reducing disease risk by fortifying natural defenses of the stock. Evidence that shows the effectiveness of probiotics in inhibiting a wide range of fish and shrimp pathogens and diseases problems in shrimp farming was also mentioned earlier in this review. Some microorganisms have been authorized for use as probiotics in feeding stuffs in the European Union. In addition, other probiotics are commercialized on the market. The last authorized list of feed additives published by the Commission [115] is tabulated in Table 5. 

## 6. Future Prospective and Concluding Remarks

The incidence of infectious diseases in shrimp aquaculture is a serious problem due to the overuse or misuse of antibiotics and antibiotic resistance genes among opportunistic pathogens such as *Vibrio* species. The application of probiotics against viruses in shrimp cultivation is a novel and safe approach. Probiotics for bacterial diseases like vibriosis is well reported but for viral diseases the authentic strains still need research. The current research is focused on the molecular interactions between the virus-probiotics and virus-host as the information available is very mere. Experiments are also under process in order to increase the potentiality of probiotic strains towards viral diseases. From the available literature and reports till date, the probiotic supplements showed better results when they are given from the starting of the culture than after the outbreak of disease. Thus, it is the best suggestion to include probiotics in the regular diet of the animal in order to prevent it from different infections and keep the animal healthy, which increases its economic value.

## Figures and Tables

**Table 1:** Viral diseases of shrimp.

Disease	Virus	Abbreviation	Genome	Family	Genus	Species affected
White spot syndrome	White spot syndrome virus	WSSV	dsDNA	Nimaviridae	Whispovirus	All farmed marine (Penaeid) shrimp species

Taura syndrome	Taura syndrome virus	TSV	(+) ssRNA	Dicistroviridae	Cripavirus	*Penaeus vannamei *

Yellow head disease	Yellow head disease virus	YHV	(+) ssRNA	Roniviridae	Okavirus	*P*.* monodon, P*.* vannamei, P*.* stylirostris *

Infectious hypodermal and hematopoietic necrosis	Infectious hypodermal and hematopoietic necrosis virus	IHHNV	ssDNA	Parvoviridae	Brevidensovirus	*P*.* monodon, P*.* vannamei, P*.* stylirostris *

Infectious myonecrosis	Infectious myonecrosis virus	IMNV	dsRNA	Totiviridae	Giardiavirus	*P*.* vannamei *

White tail disease	Macrobrachium rosenbergii nodavirus	MrNV	(+) ssRNA	Nodaviridae	Related to Alphanodavirus Betanodavirus	*Macrobrachium rosenbergii, P*.* vannamei *

**Table 2:** Indigenous microbiota of shrimp and their significance.

Name of the organism	Significance	References
Aeromonas media A199	Decrease of mortality and suppression of the pathogen of Pacific oyster larvae when challenged with a pathogenic *Vibrio tubiashii *	[24]

*Plesiomonas* spps.	Show inhibitory activity against primary pathogens *Pseudomonas aeruginosa*, *P*. *fluorescens*, secondary pathogens *Salmonella*, *Shigella*, *E. coli*, *Streptococcus *	[25]

*Pseudomonas *spp.	Potential antagonistic bacterium against pathogenic vibrios in penaeid shrimp. Produces extracellular antivibrio component	[26]

*Enterobacteriaceae *	Indicators of hygienic quality of foods and water. Their presence in prawn may be attributed to the feed or animal manure commonly used to fertilize ponds	[27]

*Vibrio* P62, *Vibrio* P63	Immunostimulatory effect in *Penaeus vannamei *	[28]
*V*. *harveyi *	Cause vibriosis in shrimp	[29]

*Bacillus *strain S11	Better yield and good control of disease and immunity enhancement in *Penaeus monodon *	[30]

Lactic acid bacteria *Lactobacillus plantarum *	Stimulate nonspecific immune response in *Litopenaeus vannamei* when challenged with *V*. *harveyi *	[31]

**Table 3:** Benefits of Probiotics in aquaculture.

Probiotic strain	Used on	Effect of probiotic strain	Reference
*Bacillus S11 *	*Penaeus monodon *	Protection against *Vibrio harveyi* by stimulation of cellular and humoral immune defenses	[30]

*Bacillus subtilis* *UTM 126 *	*Litopenaeus vannamei *	Control vibriosis by producing bacitracin, gramicidin, polymyxin, tyrotricidin, and competitive exclusion	[32]

Streptomyces	*Penaeus monodon *	Better water quality parameters, increased length and weight of the animal	[33]

*Bacillus subtilis E20 *	*Litopenaeus monodon *	Enhance humoral immune response	[34]

**Table 4:** Proteins/genes involved in WSSV antiviral defense in shrimp.

Proteins/genes involved in WSSV antiviral defense in shrimp	Species	Role in immune response	Reference
Actin	*Litopenaeus vannamei *	WSSV major structural protein VP26 binds to actin VP26-actin interaction is seen in the early stages of viral infection	[35]

ALF	*Pacifastacus leniusculus *	Interferes with WSSV replication by RNAi mechanism	[36]

*β*-Integrin	*Marsupenaeus japonicus *	Integrin mediates signal transduction and activates focal adhesion kinase (FAK). Enhance immune cell adhesion	[37]

Calreticulin	*Fenneropenaeus chinensis *	Modulate cell adhesion, phagocytosis, and integrin-dependent Ca^+2 ^signalling	[38]

Caspase-3-like gene	*Penaeus monodon *	Upregulated during WSSV infection and cause increased apoptosis	[39]

C-type lectin (CTL) (pattern recognition protein)	*Litopenaeus vannamei *	Participate in nonself-innate immune defense in invertebrates	[40]

C-type lectin (LvCTL) (mannose binding CTL)	*Litopenaeus vannamei *	Binds to WSSV envelope proteins and exert antiviral activity	[41]

Fc lectin	*Fenneropenaeus chinensis *	Enhance innate immunity, that is, immune recognition, phagocytosis	[42]

Fortilin	*Penaeus monodon *	Antiapoptotic protein, found in high levels during onset of viral infections	[43]

Hemocyanin	*Marsupenaeus japonicus *	Expression of Pj Hc, Pj HcL hemocyanin subunit genes could delay the infection to WSSV	[44]
	*Penaeus monodon *	Nonspecific antiviral properties and no cytotoxicity to host cells	[45]

LGBP	*Penaeus stylirostris *	Enhance innate immune responses, activates prophenoloxidase (proPO) case	[46]

Manganese superoxide dismutase	*Fenneropenaeus chinensis *	Enhance immune defense reactions by eliminating oxidative stress	[47]

PmAV (First antiviral gene)	*Penaeus monodon *	Inhibit virus-induced cytopathic effect	[48]

Pm CBP (chitin-binding protein)	*Penaeus monodon *	Upregulated in late stages of WSSV infection and interacts with WSSV O67C (ORF)	[49]

Pm Rab7	*Penaeus monodon *	Binds to WSSV and VP28, Inhibits WSSV-induced histopathology	[50]

Rab GTPase	*Marsupenaeus japonicas *	Upregulated in WSSV-resistant shrimp	[51]

Ran protein	*Marsupenaeus japonicus *	Upregulated in WSSV-resistant and infected shrimp	[52]

Syntenin	*Penaeus monodon *	Upregulated in acute phase of a WSSV infection	[53]

Syntenin-like protein gene	*Penaeus monodon *	Involved in signaling pathway of antiviral shrimp immune response	[54]

**Table 5:** List of microbial strains authorized as probiotics under Council Directive 70/524/EEC.

Probiotic strain	Host and applied host	Beneficial effect	Method of application	Reference
*Bacillus cereus *	*Farfantepenaeus brasiliensis *	Control vibrio concentration as well as a commercial probiotic. Mean final weight and specific growth rate of shrimp were significantly higher	Addition to culture water	[55]

*Bacillus licheniformis *	*Penaeus monodon *	Occur naturally in the intestinal tracts of prawns Compete with other bacteria in ponding and clearing the organic matter	Addition to culture water	[56]

*Bacillus subtilis *	*Penaeus monodon *	Occur naturally in the GIT. Higher shrimp growth, FCR, increased immunity to *Vibrio harveyi* 639 infection	Addition to culture water	[57]

*Saccharomyces cerevisiae *	*Litopenaeus vannamei, *	Immunostimulation and protection to *Vibrio harveyi *	Addition to diet	[58]

*Streptococcus *sp.	*Fenneropenaeus indicus *	Antagonism to *Vibrio alginolyticus *	Enrichment to live food, addition to diet	[59]

*Pediococcus acidilactici *	Artemia culture	Control *Vibrio alginolyticus* infection	Addition to diet	[60]

## References

[1] FAO The State of world fisheries and aquaculture. http://www.fao.org/docrep/005/y7300e/y7300e00.htm.

[2] FAO State of World Fisheries and Aquaculture, Food and Agricultural Organization of the United Nations. http://www.fao.org/docrep/011/i0250e/i0250e00.htm.

[3] SCAN Opinion of the Scientific Committee on Animal Nutrition on the criteria for assessing the safety of microorganisms resistant to antibiotics of human clinical and veterinary importance. http://ec.europa.eu/food/fs/sc/scan/out108_en.pdf.

[4] Naylor R, Burke M (2005). Aquaculture and ocean resources: raising tigers of the sea. *Annual Review of Environment and Resources*.

[5] Naylor RL, Eagle J, Smith WL (2003). Salmon aquaculture in the Pacific Northwest: a global industry with local impacts. *Environment*.

[6] Naylor RL, Goldburg RJ, Primavera JH (2000). Effect of aquaculture on world fish supplies. *Nature*.

[7] Goldburg R, Naylor R (2005). Future seascapes, fishing, and fish farming. *Frontiers in Ecology and the Environment*.

[8] Boxall AB, Fogg LA, Blackwell PA, Kay P, Pemberton EJ, Croxfor A (2004). Veterinary medicines in the environment. *Reviews of Environmental Contaminationand Toxicology*.

[9] Haya K, Burridge LE, Chang BD (2000). Environmental impact of chemical wastes produced by the salmon aquaculture industry. *ICES Journal of Marine Science*.

[10] Grave K, Lingaas E, Bangen M, Rønning M (1999). Surveillance of the overall consumption of antibacterial drugs in humans, domestic animals and farmed fish in Norway in 1992 and 1996. *Journal of Antimicrobial Chemotherapy*.

[11] Le TX, Munekage Y, Kato SI (2005). Antibiotic resistance in bacteria from shrimp farming in mangrove areas. *Science of the Total Environment*.

[12] Le TX, Munekage Y (2004). Residues of selected antibiotics in water and mud from shrimp ponds in mangrove areas in Viet Nam. *Marine Pollution Bulletin*.

[13] L’abée-Lund TM, Sørum H (2001). Class 1 integrons mediate antibiotic resistance in the fish pathogen *Aeromonas salmonicida* worldwide. *Microbial Drug Resistance*.

[14] Sorum H, Aarestrup FM, Washington DC (2006). Antimicrobial drug resistance in fish pathogen. *Antimicrobial Resistance in Bacteria of Animal Origin*.

[15] Angulo FJ, Nargund VN, Chiller TC (2004). Evidence of an association between use of anti-microbial agents in food animals and anti-microbial resistance among bacteria isolated from humans and the human health consequences of such resistance. *Journal of Veterinary Medicine Series B*.

[16] Cabello FC (2004). Antibiotics and aquaculture in Chile: implications for human and animal health. *Revista Medica de Chile*.

[17] Cabello FC (2003). An analysis of their potential impact upon the environment, human and animal health in Chile. *Antibiotics and Aquaculture*.

[18] Goldburg RJ, Elliot MS, Naylor RL (2001). Marine Aquaculture in the United States: environmental impacts and policy options.

[19] Grave K, Markestad A, Bangen M (1996). Comparison in prescribing patterns of antibacterial drugs in salmonid farming in Norway during the periods 1980–1988 and 1989–1994. *Journal of Veterinary Pharmacology and Therapeutics*.

[20] Davies JE, Roberts MC, Levy SB, Miller GH, Brien TF, Tenover FC (1999). Antimicrobial resistance: an ecological perspective. *A Report from the American Academy of Microbiology*.

[21] Hunter CJ, Karl D, Buckley M (2005). Marine microbial diversity: the key to earth’s habitability. *A Report from the American Academy of Microbiology*.

[22] Miranda CD, Zemelman R (2001). Antibiotic resistant bacteria in fish from the Concepción Bay, Chile. *Marine Pollution Bulletin*.

[23] Lundin CG (1996). Global attempt to address shrimp disease. *Marine Environmental Paper*.

[24] Gibson LF, Woodworth J, George AM (1998). Probiotic activity of *Aeromonas media* on the Pacific oyster, *Crassostrea gigas*, when challenged with *Vibrio tubiashii*. *Aquaculture*.

[25] Nayak SK, Mukherjee SC (2011). Screening of gastrointestinal bacteria of Indian major carps for a candidate probiotic species for aquaculture practices. *Aquaculture Research*.

[26] Rahman S, Khan SN, Naser MN, Karim MM (2011). Safety issues of isolated probiotic natured bacteria from Bangladesh coastal waters for controlling shrimp diseases. *Journal of Scientific Research*.

[27] Lalitha KV, Surendran PK (2004). Bacterial microflora associated with farmed freshwater prawn *Macrobrachium rosenbergii* (de Man) and the aquaculture environment. *Aquaculture Research*.

[28] Gullian M, Thompson F, Rodriguez J (2004). Selection of probiotic bacteria and study of their immunostimulatory effect in *Penaeus vannamei*. *Aquaculture*.

[29] Defoirdt T, Boon N, Sorgeloos P, Verstraete W, Bossier P (2007). Alternatives to antibiotics to control bacterial infections: luminescent vibriosis in aquaculture as an example. *Trends in Biotechnology*.

[30] Rengpipat S, Phianphak W, Piyatiratitivorakul S, Menasveta P (1998). Effects of a probiotic bacterium on black tiger shrimp *Penaeus monodon* survival and growth. *Aquaculture*.

[31] Vieira FN, Buglione CC, Mouriño JPL (2010). Effect of probiotic supplemented diet on marine shrimp survival after challenge with *Vibrio harveyi*. *Arquivo Brasileiro de Medicina Veterinaria e Zootecnia*.

[32] Balcázar JL, Rojas-Luna T (2007). Inhibitory activity of probiotic *Bacillus subtilis* UTM 126 against *Vibrio* species confers protection against vibriosis in juvenile shrimp (*Litopenaeus vannamei*). *Current Microbiology*.

[33] Das S, Lyla PS, Ajmal Khan S (2006). Application of *Streptomyces* as a probiotic in the laboratory culture of *Penaeus monodon (Fabricius)*. *Israeli Journal of Aquaculture*.

[34] Liu KF, Chiu CH, Shiu YL, Cheng W, Liu CH (2010). Effects of the probiotic, *Bacillus subtilis* E20, on the survival, development, stress tolerance, and immune status of white shrimp, *Litopenaeus vannamei* larvae. *Fish and Shellfish Immunology*.

[35] Xie X, Yang F (2005). Interaction of white spot syndrome virus VP26 protein with actin. *Virology*.

[36] Liu H, Jiravanichpaisal P, Söderhäll I, Cerenius L, Söderhäll K (2006). Antilipopolysaccharide factor interferes with white spot syndrome virus replication in vitro and in vivo in the crayfish *Pacifastacus leniusculus*. *Journal of Virology*.

[37] Li DF, Zhang MC, Yang HJ, Zhu YB, Xu X (2007). Beta-intergrin mediates WSSV infection. *Virology*.

[38] Luana W, Li F, Wang B, Zhang X, Liu Y, Xiang J (2007). Molecular characteristics and expression analysis of calreticulin in Chinese shrimp *Fenneropenaeus chinensis*. *Comparative Biochemistry and Physiology B*.

[39] Wongprasert K, Sangsuriya P, Phongdara A, Senapin S (2007). Cloning and characterization of a caspase gene from black tiger shrimp (*Penaeus monodon*)-infected with white spot syndrome virus (WSSV). *Journal of Biotechnology*.

[40] Ma THT, Tiu SHK, He JG, Chan SM (2007). Molecular cloning of a C-type lectin (LvLT) from the shrimp Litopenaeus vannamei: early gene down-regulation after WSSV infection. *Fish and Shellfish Immunology*.

[41] Zhao ZY, Yin ZX, Xu XP, Weng SP, Rao XY, Dai ZX (2009). A novel C-type lectin from the shrimp *Litopenaeus vannamei* possesses anti-WSSV activity. *Journal of Virology*.

[42] Liu YC, Li FH, Dong B (2007). Molecular cloning, characterization and expression analysis of a putative C-type lectin (Fclectin) gene in Chinese shrimp *Fenneropenaeus chinensis*. *Molecular Immunology*.

[43] Tonganunt M, Nupan B, Saengsakda M (2008). The role of Pm-fortilin in protecting shrimp from white spot syndrome virus (WSSV) infection. *Fish and Shellfish Immunology*.

[44] Lei K, Li F, Zhang M, Yang H, Luo T, Xu X (2008). Difference between hemocyanin subunits from shrimp *Penaeus japonicus* in anti-WSSV defense. *Developmental and Comparative Immunology*.

[45] Zhang X, Huang C, Qin Q (2004). Antiviral properties of hemocyanin isolated from shrimp *Penaeus monodon*. *Antiviral Research*.

[46] Roux MM, Pain A, Klimpel KR, Dhar AK (2002). The lipopolysaccharide and *β*-1,3-glucan binding protein gene is upregulated in white spot virus-infected shrimp (*Penaeus stylirostris*). *Journal of Virology*.

[47] Zhang Q, Li F, Wang B (2007). The mitochondrial manganese superoxide dismutase gene in Chinese shrimp *Fenneropenaeus chinensis*: Cloning, distribution and expression. *Developmental and Comparative Immunology*.

[48] Luo T, Zhang X, Shao Z, Xu X (2003). PmAV, a novel gene involved in virus resistance of shrimp *Penaeus monodon*. *FEBS Letters*.

[49] Chen LL, Lu LC, Wu WJ, Lo CF, Huang WP (2007). White spot syndrome virus envelope protein VP53A interacts with *Penaeus monodon* chitin-binding protein (PmCBP). *Diseases of Aquatic Organisms*.

[50] Sritunyalucksana K, Wannapapho W, Lo CF, Flegel TW (2006). PmRab7 is a VP28-binding protein involved in white spot syndrome virus infection in shrimp. *Journal of Virology*.

[51] Wu W, Zhang X (2007). Characterization of a Rab GTPase up-regulated in the shrimp *Peneaus japonicus* by virus infection. *Fish and Shellfish Immunology*.

[52] Han F, Zhang X (2007). Characterization of a ras-related nuclear protein (Ran protein) up-regulated in shrimp antiviral immunity. *Fish and Shellfish Immunology*.

[53] Tonganunt M, Phongdara A, Chotigeat W, Fujise K (2005). Identification and characterization of syntenin binding protein in the black tiger shrimp *Penaeus monodon*. *Journal of Biotechnology*.

[54] Bangrak P, Graidist P, Chotigeat W, Supamattaya K, Phongdara A (2002). A syntenin-like protein with postsynaptic density protein (PDZ) domains produced by black tiger shrimp *Penaeus monodon* in response to white spot syndrome virus infection. *Diseases of Aquatic Organisms*.

[55] Moreira D, Sabrina M, Leivas FP, Romano LA, Luis E, Ballester E (2011). *New Bacillus Probiotic Tested for Shrimp*.

[56] Moriarty DJW, Decamp OP (2005). *Probiotics in Aquaculture*.

[57] Utiswannakul P, Sangchai S, Rengpipat S (2011). Enhanced growth of black tiger shrimp *Penaeus monodon* by dietary supplementation with *Bacillus* (BP11) as a probiotic. *Journal of Aquatic Research and Development*.

[58] Scholz U, Garcia Diaz G, Ricque D, Cruz Suarez LE, Vargas Albores F, Latchford J (1999). Enhancement of vibriosis resistance in juvenile *Penaeus vannamei* by supplementation of diets with different yeast products. *Aquaculture*.

[59] Ajitha S, Sridhar M, Sridhar N, Singh ISB, Varghese V (2004). Probiotic effects of lactic acid bacteria against *Vibrio alginolyticus* in *Penaeus (Fenneropenaeus)* indicus. *Asian Journal of Fishery Sciences*.

[60] Villamil L, Figueras A, Planas M, Novoa B (2003). Control of *Vibrio alginolyticus* in Artemia culture by treatment with bacterial probiotics. *Aquaculture*.

[61] Bondad-Reantaso MG, Subasinghe RP, Arthur JR (2005). Disease and health management in Asian aquaculture. *Veterinary Parasitology*.

[62] Norasma D, Saleem M (2008). *Effluent and Disease Management in Traditional Practices of Shrimp Farming: A Case Study on the West Coast of Sabah, Malaysia*.

[63] PÁez-Osuna F (2001). The environmental impact of shrimp aquaculture: causes, effects, and mitigating alternatives. *Environmental Management*.

[64] Karunasagar I, Shivu MM, Girisha SK, Krohne G, Karunasagar I (2007). Biocontrol of pathogens in shrimp hatcheries using bacteriophages. *Aquaculture*.

[65] Fuller R (1997). *Probiotics 2: Applications and Practical Aspects*.

[66] Fuller R (1992). *Probiotics: The Scientific Basis*.

[67] Fuller R (1989). Probiotics in man and animals. *Journal of Applied Bacteriology*.

[68] Tannock GW, Fuller R, Pedersen K (1999). Lactobacillus succession in the piglet digestive tract demonstrated by plasmid profiling. *Applied and Environmental Microbiology*.

[69] FAO/WHO (2001). Report of a Joint FAO/WHO expert consultation on evaluation of health and nutritional propeties of probiotics in food including powder milk with live lactic acid bacteria. *Health and Nutritional Properties of Probiotics in Food Including Powder Milk with Live Lactic Acid Bacteria*.

[70] Verschuere L, Rombaut G, Sorgeloos P, Verstraete W (2000). Probiotic bacteria as biological control agents in aquaculture. *Microbiology and Molecular Biology Reviews*.

[71] Onarheim AM, Wiik R, Burghardt J, Stagkebrandt E (1994). Characterization and identification of two *Vibrio* species indigenous to the intestine of fish in cold sea water; description of *Vibrio iliopiscarius* sp. nov.. *Systematic and Applied Microbiology*.

[72] Sakata T, Lesel R (1990). Microflora in the digestive tract of fish and shell-fish. *Microbiology in Poecilotherms*.

[73] Ringø E, Vadstein O (1998). Colonization of *Vibrio pelagius* and *Aeromonas caviae* in early developing turbot (*Scophthalmus maximus* L.) larvae. *Journal of Applied Microbiology*.

[74] Austin B, Baudet E, Stobie M (1992). Inhibition of bacterial fish pathogens by *Tetraselmis suecica*. *Journal of Fish Diseases*.

[75] Austin B, Stuckey LF, Robertson PAW, Effendi I, Griffith DRW (1995). A probiotic strain of *Vibrio alginolyticus* effective in reducing diseases caused by *Aeromonas salmonicida*, *Vibrio anguillarum* and *Vibrio ordalii*. *Journal of Fish Diseases*.

[76] Douillet PA, Langdon CJ (1994). Use of a probiotic for the culture of larvae of the Pacific oyster (*Crassostrea gigas* Thunberg). *Aquaculture*.

[77] Gildberg A, Johansen A, Bogwald J (1995). Growth and survival of Atlantic salmon (*Salmo salar*) fry given diets supplemented with fish protein hydrolysate and lactic acid bacteria during a challenge trial with *Aeromonas salmonicida*. *Aquaculture*.

[78] Gildberg A, Mikkelsen H, Sandaker E, Ringø E (1997). Probiotic effect of lactic acid bacteria in the feed on growth and survival of fry of Atlantic cod (*Gadus morhua*). *Hydrobiologia*.

[79] Phianphak W, Rengpipat S, Piyantiratitivorakul S, Menasveta P (1999). Probiotic use of *Lactobacillus* spp. for black tiger shrimp. *Penaeus monodon*. *Journal of Scientific Research, Chulanokorn University*.

[80] Austin B, Day JG (1990). Inhibition of prawn pathogenic *Vibrio* spp. by a commercial spray-dried preparation of *Tetraselmis suecica*. *Aquaculture*.

[81] Austin B, Billaud AC (1990). Inhibition of fish pathogen, *Serratia liquefaciens*, By an antibiotic producing isolate of *Plamococcus* recovered from sea water. *Journal of Fish Diseases*.

[82] Dopazo CP, Lemos ML, Lodeiros C, Bolinches J, Barja JL, Toranzo AE (1988). Inhibitory activity of antibiotic-producing marine bacteria against fish pathogens. *Journal of Applied Bacteriology*.

[83] Munro PD, McLean HA, Barbour A, Birkbeck TH (1995). Stimulation or inhibition of growth of the unicellular alga *Pavlovalutheri* by bacteria isolated from larval turbot culture systems. *Journal of Applied Bacteriology*.

[84] Westerdahl A, Olsson JC, Kjelleberg S, Conway PL (1991). Isolation and characterization of turbot (*Scophtalmus maximus*)-associated bacteria with inhibitory effects against *Vibrio anguillarum*. *Applied and Environmental Microbiology*.

[85] Quigley EMM (2010). Prebiotics and probiotics; modifying and mining the microbiota. *Pharmacological Research*.

[86] Sherman PM, Ossa JC, Johnson HK (2009). Unravelling mechanisms of action of probiotics. *Nutrition Clinical Practices*.

[87] Mayra MA, Bigret M, Salminen SV, Wright A, Marcel D (1993). Industrial use and production of lactic acid bacteria. *Lactic Acid Bacteria*.

[88] Yasuds K, Taga NA (1980). Mass culture method for *Artemia salina* using bacteria as food. *Medical Education Resources*.

[89] Moriarty DJW (1997). The role of microorganisms in aquaculture ponds. *Aquaculture*.

[90] Karunasagar I, Otta SK, Karunasagar I, Joshua K (1996). Applications of *Vibrio* vaccine in shrimp culture. *Fishing Chimes*.

[91] Soundarapandian P, Ramanan V, Dinakaran GK (2010). Effect of probiotics on the growth and survival of *Penaeus monodon* (Fabricius). *Current Research Journal of Social Sciences*.

[92] Holzapfel WH, Haberer P, Snel J, Schillinger U, Huisss JHJ (1998). Overview of gut flora and probiotics. *International Journal of Food Microbiology*.

[93] Chiu CH, Guu YK, Liu CH, Pan TM, Cheng W (2007). Immune responses and gene expression in white shrimp, *Litopenaeus vannamei*, induced by *Lactobacillus plantarum*. *Fish and Shellfish Immunology*.

[94] Castex M, Lemaire P, Wabete N, Chim L (2010). Effect of probiotic *Pediococcus acidilactici* on antioxidant defences and oxidative stress of *Litopenaeus stylirostris* under *Vibrio nigripulchritudo* challenge. *Fish and Shellfish Immunology*.

[95] Thompson J, Gregory S, Plummer S, Shields RJ, Rowley AF (2010). An in vitro and in vivo assessment of the potential of *Vibrio* spp. as probiotics for the Pacific White shrimp, *Litopenaeus vannamei*. *Journal of Applied Microbiology*.

[96] Roch P (1999). Defense mechanisms and disease prevention in farmed marine invertebrates. *Aquaculture*.

[97] Jiravanichpaisal P, Lee BL, Söderhäll K (2006). Cell-mediated immunity in arthropods: hematopoiesis, coagulation, melanization and opsonization. *Immunobiology*.

[98] Lee KK, Liu PC, Kou GH, Chen SN (1997). Passive immunization of the tiger prawn, *Penaeus monodon*, using rabbit antisera to *Vibrio harveyi*. *Letters in Applied Microbiology*.

[99] Witteveldt J, Cifuentes CC, Vlak JM, Van Hulten MCW (2004). Protection of *Penaeus monodon* against white spot syndrome virus by oral vaccination. *Journal of Virology*.

[100] Rout N, Kumar S, Jaganmohan S, Murugan V (2007). DNA vaccines encoding viral envelope proteins confer protective immunity against WSSV in black tiger shrimp. *Vaccine*.

[101] Kawai T, Akira S (2006). Innate immune recognition of viral infection. *Nature Immunology*.

[102] Haipeng L, Soderhall K, Jiranvanichpaisal P (2009). Antiviral immunity in Crustaceans. *Fish and Shellfish Immunology*.

[103] Wang W, Zhang X (2008). Comparison of antiviral efficiency of immune responses in shrimp. *Fish and Shellfish Immunology*.

[104] Liu W, Han F, Zhang X (2009). Ran GTPase regulates hemocytic phagocytosis of shrimp by interaction with myosin. *Journal of Proteome Research*.

[105] Wu W, Wang L, Zhang X (2005). Identification of white spot syndrome virus (WSSV) envelope proteins involved in shrimp infection. *Virology*.

[106] Xu J, Han F, Zhang X (2007). Silencing shrimp white spot syndrome virus (WSSV) genes by siRNA. *Antiviral Research*.

[107] Ting Y, Rangrong Z, Xiaobo Z (2012). The role of White spot syndrome virus (WSSV) VP466 protein in shrimp antiviral phagocytosis. *Fish and Shellfish Immunology*.

[108] Xu J, Han F, Zhang X (2007). Silencing shrimp white spot syndrome virus (WSSV) genes by siRNA. *Antiviral Research*.

[109] Kamei Y, Yoshimizu M, Ezura Y, Kimura T (1988). Screening of bacteria with antiviral activity from fresh water salmonid hatcheries. *Microbiology and Immunology*.

[110] Direkbusarakom S, Yoshimizu M, Ezura Y, Ruangpan L, Danayadol Y (1998). Vibrio spp., the dominant flora in shrimp hatchery against some fish pathogenic viruses. *Journal of Marine Biotechnology*.

[111] Botić T, Klingberg TD, Weingartl H, Cencič A (2007). A novel eukaryotic cell culture model to study antiviral activity of potential probiotic bacteria. *International Journal of Food Microbiology*.

[112] Isolauri E (2003). Probiotics for infectious diarrhoea. *Gut*.

[113] George SK, Kaizer KN, Betz YM, Dhar AK (2011). Multiplication of Taura syndrome virus in primary hemocyte culture of shrimp (*Penaeus vannamei*). *Journal of Virological Methods*.

[114] Otoshi CA, Arce SM, Moss SM (2003). Growth and reproductive performance of broodstock shrimp reared in a biosecure recirculating aquaculture system versus a flow-through pond. *Aquacultural Engineering*.

[115] Council Directive 70/524/EEC (2004). List of the authorized additives in feeding stuffs published in application of Article 9t of Council Directive 70/554/EEC concerning additives in feedingstuffs. *Official Journal of European Union*.

